# Supplementary figures and images for: Investigating Ecological Momentary Assessed Physical Activity and Core Executive Functions in 18- to 24-Year-Old Undergraduate Students
Source: Int J Environ Res Public Health. 2023 Oct 19;20(20):6944. doi: 10.3390/ijerph20206944 (PMC10606779; doi:10.3390/ijerph20206944)

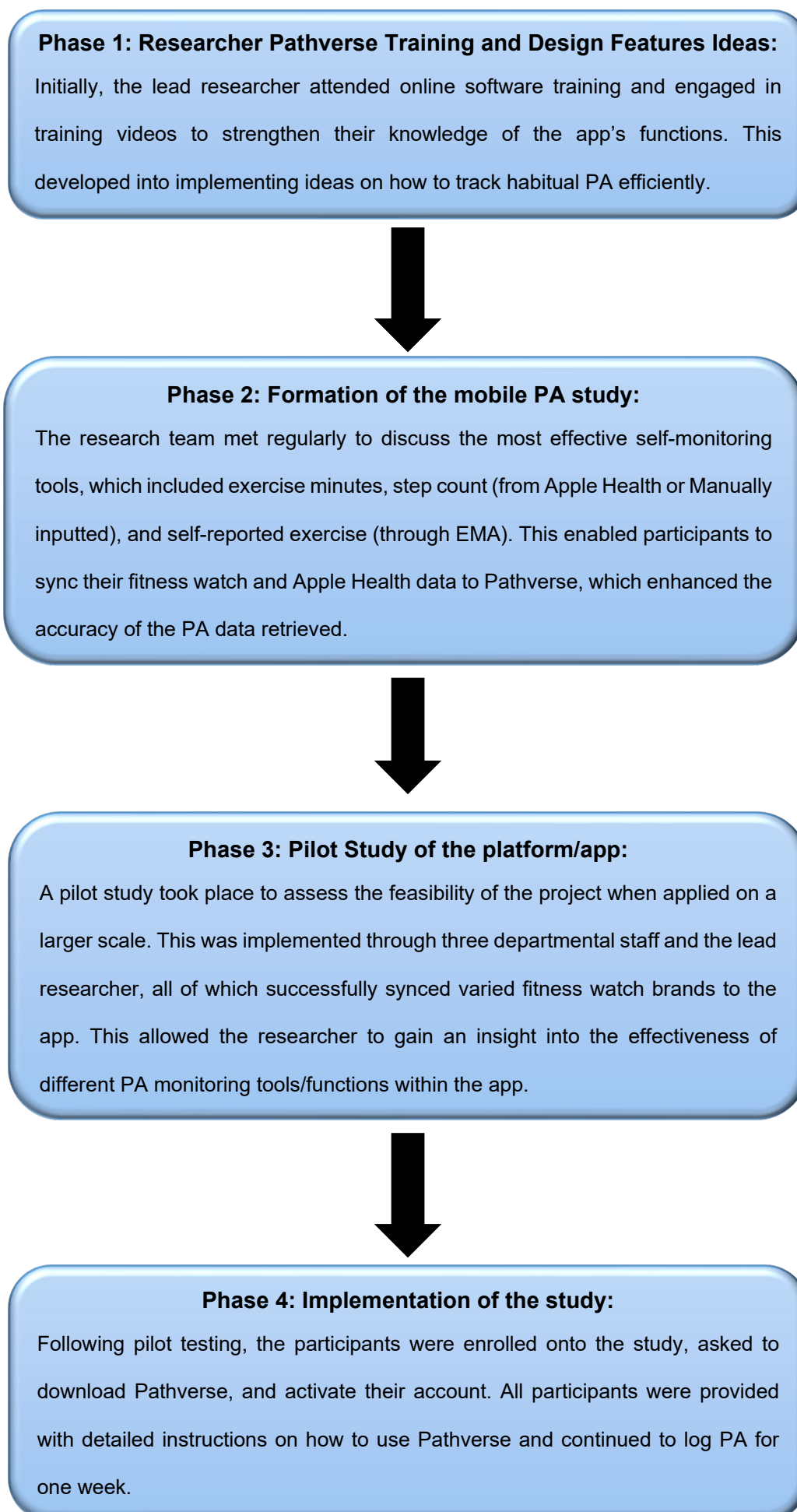

**Figure S1.** Pathverse Online Study Development Phases.

Supplement: Supplementary file 1 [file ijerph-20-06944-s001.zip › ijerph-2640478-supplementary.pdf]
